# Effects of Pender’s Health Promotion Model-Based Online Education and Telephone Follow-Up on Symptom Burden, Dietary Behaviors, and Medication Adherence in Adults with Heart Failure: A Randomized Controlled Trial

**DOI:** 10.3390/healthcare14213796

**Published:** 2026-11-06

**Authors:** Ahmet Enes Güngördü, Naile Alankaya, Sevil Albayrak

**Affiliations:** 1 Department of Nursing, Graduate School of Education and Research, Cyprus Aydin University, 99320 Kyrenia, Cyprus; 2 Department of Nursing, Faculty of Health Sciences, Çanakkale Onsekiz Mart University, 17100 Canakkale, Türkiye; naile.alankaya@gmail.com; 3 Department of Nursing, Faculty of Health Sciences, Cyprus Aydin University, 99320 Kyrenia, Cyprus; sevilalbayrak@cau.edu.tr

**Keywords:** heart failure, Pender’s Health Promotion Model, online education, telephone follow-up, self-management, nursing intervention

## Abstract

**Background/Objectives:** Effective heart failure (HF) self-management requires sustained engagement in symptom management, dietary behaviors, and medication adherence. This randomized controlled trial evaluated Pender’s Health Promotion Model (HPM)-informed online education combined with telephone reinforcement in adults with HF. **Methods:** In this single-center, parallel-group trial, 60 adults were randomized 1:1 to intervention (*n* = 30) or usual care (*n* = 30). The intervention comprised individualized synchronous online education and telephone reinforcement at 2 weeks and 1 month. Self-reported dietary behaviors, symptom burden, and medication adherence were assessed at baseline and 2 months. Linear mixed-effect models evaluated group-by-time effects, with Holm adjustment for three prespecified primary outcomes. Age-adjusted sensitivity analyses were conducted. The trial was prospectively registered (NCT07017751). **Results:** All 60 participants completed follow-up. Group-by-time effects favored the intervention for all outcomes (Holm-adjusted *p* < 0.001 each). Between-group differences in change were 12.77 points (95% CI, 10.22 to 15.31) for dietary behaviors, −17.27 (95% CI, −24.56 to −9.97) for symptom burden, and −2.33 (95% CI, −3.37 to −1.30) for medication adherence. Findings remained significant after age adjustment. **Conclusions:** HPM-informed online education combined with telephone reinforcement improved self-reported dietary behaviors and medication adherence and reduced symptom burden compared with usual care over 2 months. Although the unblinded design and reliance on self-report warrant cautious interpretation, these findings support the potential value of this combined nursing intervention for short-term HF self-management. Larger trials with longer follow-up and objective clinical outcomes are needed.

## 1. Introduction

Heart failure (HF) is a complex chronic condition that requires substantial day-to-day self-care alongside guideline-directed medical treatment. Contemporary HF guidelines conceptualize self-care as encompassing maintenance behaviors, symptom monitoring, and appropriate responses to signs and symptoms of clinical deterioration [1,2]. Medication adherence and appropriate dietary behaviors are central components of self-care, while recognizing and monitoring changes in HF symptoms is essential for the timely identification and management of worsening clinical status [1,2]. Nevertheless, effective self-care can be challenging because HF-related behaviors are influenced by interacting health-related, psychosocial, sociocultural, economic, and knowledge-related factors [3]. Accordingly, comprehensive HF management extends beyond pharmacological treatment and requires patient education and ongoing support to facilitate the integration of symptom monitoring and recommended medication- and diet-related behaviors into daily life [1].

Nurses are well positioned to support HF self-care through structured education, behavioral reinforcement, symptom monitoring, and continued follow-up. Recent evidence suggests that nursing interventions may improve selected dimensions of HF self-care, particularly when education is combined with follow-up, motivational support, or practical monitoring resources, although the magnitude and consistency of effects vary across interventions and follow-up periods [4]. Similarly, a systematic review and meta-analysis of nine randomized controlled trials involving 952 patients with HF found that nurse-led, technology-based interventions improved self-care maintenance, self-management, and self-confidence; seven of the nine trials incorporated telephone-based support [5]. However, substantial between-study heterogeneity was observed, reflecting variation in settings, populations, technologies, and follow-up approaches [5]. Digital approaches combining education with remote follow-up have also been investigated specifically in HF. For example, a randomized controlled trial of 64 patients found that web-based education combined with telephone follow-up and weekly text messages improved symptom burden and health status compared with routine care [6]. More recently, a systematic review and meta-analysis of 26 randomized controlled trials found potential clinical benefits of digital health interventions incorporating educational components, while also demonstrating considerable variation in intervention modality, intensity, duration, comparator care, and follow-up [7]. Most interventions combined education with additional components such as telemonitoring, clinician feedback, symptom tracking, or remote disease management, making it difficult to isolate the contribution of individual intervention components [7]. These findings underscore the need for clearly defined interventions in which the theoretical basis, educational content, mode of delivery, and follow-up strategy are explicitly described.

Theory-based interventions may provide a systematic approach to supporting the complex behavioral changes required for effective HF self-care. Pender’s Health Promotion Model (HPM) considers individual characteristics and experiences together with behavior-specific cognitions and affect, including perceived benefits of action, perceived barriers to action, perceived self-efficacy, interpersonal influences, and situational influences, which contribute to commitment to a plan of action and subsequent health-promoting behavior [8]. These constructs provide a useful framework for addressing behaviors that patients with HF are expected to incorporate into daily life, including dietary practices, medication-taking behaviors, and responses to symptoms [8,9]. Previous cardiovascular research supports the application of the HPM in structured nursing interventions. Among patients with HF, Habibzadeh et al. [9] reported that an HPM-based educational intervention improved several dimensions of health-promoting behavior and quality of life compared with the control condition. Similarly, a nurse-led HPM-based randomized controlled trial among patients with coronary artery disease demonstrated improvements in exercise-related self-efficacy and exercise behavior [10]. Nevertheless, despite evidence supporting digital and telephone-based HF education and separate evidence supporting HPM-based cardiovascular interventions, evidence remains limited regarding theory-driven HF interventions that integrate structured online education with planned telephone reinforcement while simultaneously evaluating symptom burden and specific dietary and medication-related behaviors.

Against this background, the present study evaluated a theory-driven nursing intervention combining structured online education based on Pender’s Health Promotion Model with planned telephone follow-up and reinforcement. Rather than focusing solely on general self-care or quality of life, the intervention targeted three interrelated aspects of HF management: symptom burden, dietary behaviors, and medication adherence. Accordingly, this randomized controlled trial aimed to determine the effects of Pender’s Health Promotion Model-based structured online education combined with planned telephone follow-up on symptom burden, dietary behaviors, and medication adherence among adults with HF. HF symptom burden, HF-related dietary behaviors, and medication adherence were evaluated as the three prespecified primary outcomes. It was hypothesized that, compared with participants receiving routine clinical care, participants receiving the intervention would demonstrate (H1) a greater reduction in HF symptom burden over time, (H2) greater improvement in HF-related dietary behaviors over time, and (H3) greater improvement in medication adherence over time.

## 2. Materials and Methods

### 2.1. Study Design and Setting

**Figure 1:**
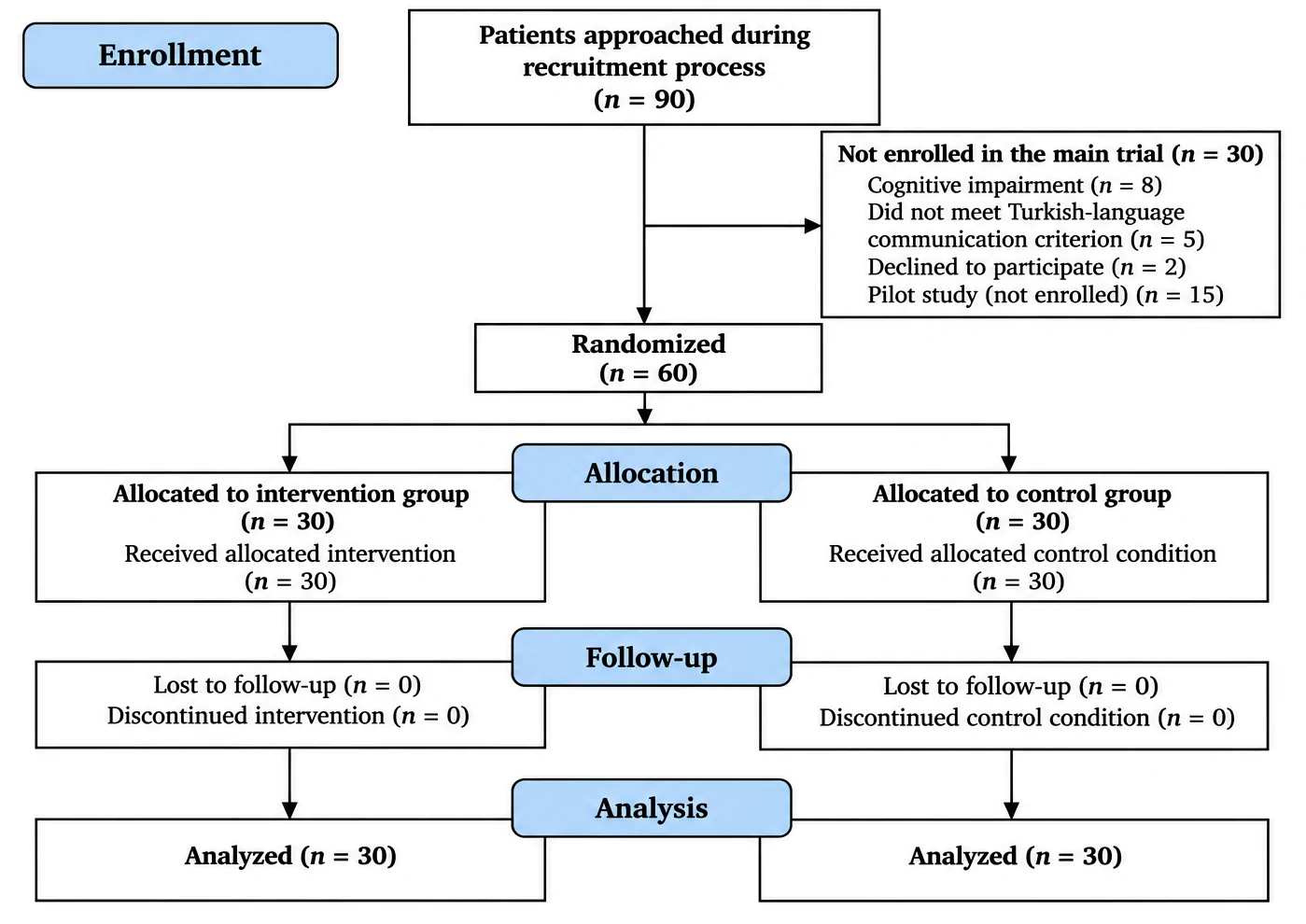
CONSORT flow diagram of participant enrollment, allocation, follow-up, and analysis.

This study was conducted as a two-arm, parallel-group randomized controlled trial at Dr. Burhan Nalbantoğlu State Hospital in the Turkish Republic of Northern Cyprus. Participants were recruited from the cardiology and internal medicine outpatient clinics and the inpatient cardiology ward. Data collection for the main trial was conducted between 20 December 2025 and 30 March 2026. The trial was prospectively registered at ClinicalTrials.gov (NCT07017751; first posted on 11 June 2025) before enrollment of the first participant. No substantive changes to the prespecified trial methods were made after commencement of participant recruitment. Before recruitment to the main study, the educational intervention was pilot-tested to assess the clarity, feasibility, and delivery of the educational content and subsequently refined in 15 patients who were not included in the main study or final analyses. This randomized controlled trial was reported in accordance with the CONSORT 2025 statement (Consolidated Standards of Reporting Trials). The participant flow is presented in Figure 1. 

### 2.2. Participants and Recruitment

Participants were eligible if they were aged ≥18 years; had a confirmed diagnosis of heart failure established by a cardiologist; had the technical resources required to participate in the online education and telephone follow-up, including access to a smartphone, tablet, or computer with an active internet connection; were literate and able to read, understand, and communicate in written Turkish; and provided written informed consent. Participants were excluded if they had severe cognitive impairment or an active psychiatric disorder that could interfere with data collection, education, or follow-up; severe visual, hearing, or speech impairment that prevented the verbal or written communication required for study participation; or were on a heart transplantation waiting list or had recently undergone heart transplantation and were under post-transplant follow-up.

Potentially eligible participants were recruited from the cardiology and internal medicine outpatient clinics and the inpatient cardiology ward of Dr. Burhan Nalbantoğlu State Hospital. In the outpatient clinics, patients with a confirmed diagnosis of HF were referred to the researchers by their treating physicians and were subsequently assessed for eligibility. Among hospitalized patients, potentially eligible individuals were identified based on a documented HF diagnosis in their medical records and were then assessed by the researchers against the eligibility criteria.

A total of 90 patients were approached during the recruitment process. Eight were ineligible because of cognitive impairment, five did not meet the Turkish-language communication criterion, and two declined participation in the online intervention. An additional 15 eligible patients participated in the pilot phase and were not enrolled in the main randomized trial. The remaining 60 participants provided written informed consent and were enrolled in the main trial.

### 2.3. Sample Size

The sample size calculation was informed by a previous pilot randomized controlled trial conducted by Athilingam et al. (2017) [11], which evaluated a mobile health intervention targeting self-care outcomes in patients with heart failure. That study reported a relatively large effect for self-care management (partial η^2^ = 0.249). Self-care management was considered conceptually relevant to the present study because dietary behaviors and medication adherence are important components of heart failure self-management. However, the interventions and outcome measures differed between the two studies.

For sample size planning, a large standardized effect size (Cohen’s d = 1.128) was assumed. An a priori power analysis was performed using G*Power version 3.1.9.7 for a two-sided independent-samples *t*-test, with a significance level of 0.05, statistical power of 95%, and equal group allocation. The minimum required sample size was 44 participants (22 per group). To account for potential attrition, the target sample size was increased to 60 participants (30 per group). The calculation was based on the assumed effect size rather than separate power calculations for each of the three prespecified primary outcomes.

### 2.4. Randomization and Allocation Concealment

After eligibility was confirmed and written informed consent was obtained, participants completed baseline (T0) assessments before being randomly assigned in a 1:1 ratio to the intervention or control group using a computer-generated randomization sequence prepared by an independent individual. Group assignments were placed in sealed envelopes prepared by an independent individual. The researcher remained unaware of group allocation during baseline assessments. After completion of the baseline assessment for each participant, the researcher opened the corresponding sealed envelope in the participant’s presence, revealing the group assignment.

Because of the nature of the educational intervention, participants and the researcher delivering the intervention could not be blinded to group allocation. Statistical analyses were performed by an analyst who was blinded to treatment allocation. Study groups were identified using coded labels, and the analyst remained unaware of which code corresponded to the intervention or control group during the analyses.

### 2.5. Intervention and Control Conditions

#### 2.5.1. Intervention Group

Participants allocated to the intervention group received an individually delivered, synchronous online educational intervention structured according to Pender’s Health Promotion Model (HPM). The education was delivered in a single session scheduled at a mutually convenient date and time according to each participant’s availability. Sessions were conducted via online communication platforms, and participants joined using a smartphone or computer depending on their available technology and preference. Each educational session lasted approximately 25–30 min. Participants in the intervention group continued to receive routine clinical care throughout the study, with the HPM-based online education and planned telephone follow-up provided in addition to usual care.

The education was delivered individually by the researcher using a standardized PowerPoint presentation developed by the research team. The educational material was reviewed by experts and finalized following pilot testing before its use in the main randomized trial. Following a brief introduction to HF, the intervention focused on the three target areas of the study: HF-related dietary behaviors, symptom management, and medication adherence. These areas were addressed using HPM-based components, including behavior-specific perceptions and influences, perceived benefits, perceived barriers, perceived self-efficacy, motivation and readiness for change, and available sources of support [8]. Following the online session, the educational material was shared with participants in digital format to enable subsequent review.

Following the online education session, the researcher conducted two planned telephone follow-up calls, approximately 2 weeks and 1 month after the education. During these calls, the researcher assessed participants’ current HF-related symptoms, dietary practices, and medication use. Participants were given an opportunity to discuss difficulties or questions related to these areas. When additional information was needed, the researcher provided individualized clarification and reinforced the relevant educational content by telephone. Thus, the telephone contacts functioned as a planned reinforcement component of the intervention rather than solely as outcome-assessment contacts.

The online educational sessions and telephone follow-up contacts were delivered by the same researcher. A structured participant-specific follow-up form was used to document the timing and duration of each telephone contact, participants’ questions and difficulties, and the individualized information provided in response. The completed follow-up forms were subsequently reviewed by two co-investigators to monitor the implementation of the telephone reinforcement component. The telephone sessions were not directly observed, and no independent fidelity assessment was conducted.

Outcome assessments were conducted at baseline (T0), before group allocation, and again 2 months after the baseline assessment (T1). The telephone contacts at 2 weeks and 1 month were components of the intervention and were not formal outcome-assessment time points.

#### 2.5.2. Control Group

Participants allocated to the control group did not receive the structured HPM-based education or planned telephone follow-up from the research team during the study period. They continued to receive routine clinical care at the hospital. This included ongoing contact with physicians and nurses through outpatient or inpatient services, as applicable, during which patients could receive information and advice regarding medication use, dietary practices, and other aspects of HF care. The content and frequency of routine counseling were not standardized or systematically documented by the research team, and the existence of a hospital-wide standardized patient education protocol could not be verified. Participants in the control group completed the same outcome assessments as the intervention group at baseline and 2 months after baseline. After completion of the final outcome assessment, they were provided with a printed version of the educational material used in the intervention group.

### 2.6. Outcome Measures

The three prespecified primary outcomes were HF symptom burden, HF-related dietary behaviors, and medication adherence. These outcomes were assessed using the Heart Failure Symptom Status Scale (HFSSS), the Heart Failure Dietary Behavior Scale (HFDBS), and the 7-item Adherence to Refills and Medications Scale (ARMS-7), respectively. All three outcomes were assessed at baseline (T0) and 2 months after baseline (T1). Harms or unintended effects were not prespecified as study outcomes and were not systematically assessed.

#### 2.6.1. Heart Failure Symptom Status Scale

Heart failure symptom burden was assessed using the Heart Failure Symptom Status Scale (HFSSS), originally developed by Heo et al. [12] and subsequently adapted and psychometrically evaluated for use in Turkish by Gök Metin and Gülbahar [13]. The scale assesses common HF-related symptoms, including dyspnea, orthopnea, fatigue, chest pain, edema, sleep difficulties, and dizziness/balance-related symptoms, in terms of symptom frequency, severity, and associated distress. Symptom frequency is rated from 1 (less than once a week) to 4 (almost every day), symptom severity from 1 (mild) to 4 (very severe), and symptom-related distress from 0 (not at all) to 4 (very much). If a symptom is not experienced, it contributes 0 points to the score. The initial yes/no indicator used to establish the presence of a symptom is not included as an additional point in the total score. The total score ranges from 0 to 84, with higher scores indicating greater HF symptom burden. The original scale demonstrated a Cronbach’s α of 0.80, while the Turkish adaptation reported a Cronbach’s α of 0.86. In the present study, Cronbach’s α for the total scale was 0.935 at baseline and 0.956 at the 2-month assessment.

#### 2.6.2. Heart Failure Dietary Behavior Scale

HF-specific dietary behaviors were assessed using the Heart Failure Dietary Behavior Scale (HFDBS), developed by Boy and Enç [14]. The scale consists of 19 items grouped into four subscales: healthy habits (items 4, 5, 11, 12, and 16), salt restriction (items 1, 3, 7, 9, 10, and 15), sugar restriction (items 6, 17, and 19), and prevention of fluid retention (items 2, 8, 13, 14, and 18). Items are rated on a four-point Likert scale ranging from 1 (never) to 4 (always). Items 3, 6, 7, 9, 10, 15, and 19 are negatively worded and are reverse-scored before calculation of the total score. The item scores are summed to obtain a total score ranging from 19 to 76, with higher scores indicating greater adherence to HF-specific dietary recommendations. In the original development study, the scale demonstrated a Cronbach’s α of 0.72; factor loadings ranged from 0.473 to 0.910, and the four-factor structure explained 48.42% of the total variance [14]. In the present study, Cronbach’s α for the total scale was 0.599 at baseline and 0.819 at the 2-month assessment.

#### 2.6.3. Adherence to Refills and Medications Scale

Medication adherence was assessed using the 7-item Adherence to Refills and Medications Scale (ARMS-7), a Turkish adaptation of the Adherence to Refills and Medications Scale originally developed by Kripalani et al. [15]. The Turkish ARMS-7 was adapted and psychometrically evaluated by Gökdoğan and Kes [16]. The scale consists of seven items rated on a four-point Likert scale ranging from 1 (never) to 4 (always) and comprises two subscales: medication adherence (four items; score range, 4–16) and prescription refill (three items; score range, 3–12). Item 7 is reverse-scored before calculation of the scale scores. The seven item scores are summed to obtain a total score ranging from 7 to 28. Lower total scores indicate better medication adherence, whereas higher scores indicate poorer adherence. The Turkish version demonstrated a Cronbach’s α of 0.75 [16]. In the present study, Cronbach’s α for the total scale was 0.734 at baseline and 0.758 at the 2-month assessment.

### 2.7. Data Collection

Baseline data (T0) were collected after eligibility was confirmed and written informed consent was obtained, but before group allocation. At the time of the baseline assessment, the researcher was unaware of the participant’s subsequent group assignment. Participants completed the baseline questionnaires themselves using paper-based forms at the hospital, either in the relevant outpatient clinic or, for hospitalized participants, in the inpatient cardiology ward. Sociodemographic information and clinical information known by the participants were self-reported, whereas the diagnosis of HF was verified from the medical records. NYHA functional class was recorded when the information was available from the participant or could be verified from the medical records. When participants were unable to report their NYHA class and the classification was not documented in the available medical records, it was categorized as “Unknown”.

After completion of all baseline assessments, the sealed allocation envelope was opened by the researcher in the presence of the participant, and the group assignment was revealed. Participants allocated to the intervention group subsequently received the HPM-based online education and the two planned telephone follow-up contacts at 2 weeks and 1 month. All 30 participants allocated to the intervention group received the educational session and completed both planned telephone follow-up contacts.

Follow-up outcome data (T1) were collected 2 months after each participant’s individual baseline assessment date. To reduce the burden of returning to the hospital solely for research assessments, the same questionnaires were transferred to Google Forms for the 2-month follow-up. The follow-up questionnaires were administered electronically using Google Forms, with the questionnaire link sent through the contact information provided by the participant, including e-mail or telephone-based communication. Participants completed the questionnaires electronically and independently, without direct researcher assistance or supervision. Minor scheduling variations of approximately 1–2 days around the planned 2-month assessment occurred for some participants. The researcher was aware of group allocation at T1; therefore, follow-up outcome assessment was not blinded.

All 60 randomized participants completed the 2-month follow-up assessment, with no loss to follow-up. Accordingly, complete baseline and follow-up outcome data were available for all 30 participants in the intervention group and all 30 participants in the control group.

Patient and public involvement: patients and the public were not involved in this study beyond their participation as research participants.

### 2.8. Statistical Analysis

Statistical analyses were performed using IBM SPSS Statistics for Windows, version 25.0 (IBM Corp., Armonk, NY, USA). Additional age-adjusted mixed-effect analyses were performed using Python (statsmodels, version 0.14.6). Descriptive statistics were used to summarize participant characteristics and study variables. Continuous variables are presented as means and standard deviations (SDs), and categorical variables as frequencies and percentages. The internal consistency of the three outcome measures was evaluated separately at baseline and at the 2-month assessment using Cronbach’s alpha (α) coefficients. Data entry and statistical analyses were performed using coded group identifiers, such that group allocation was masked during these procedures.

The effects of the intervention on the three prespecified outcomes—HF symptom burden, HF-related dietary behaviors, and medication adherence—were evaluated using separate linear mixed-effect models (LMMs) based on the total score of each outcome measure. Each model included group (intervention vs. control), time (baseline vs. 2 months), and the group × time interaction as fixed effects, with participant included as a random effect to account for the correlation between repeated measurements within individuals. The group × time interaction was the principal test of the intervention effect and indicated whether the magnitude of change from baseline to 2 months differed between the intervention and control groups.

To account for multiple testing across the three prespecified primary outcomes, the Holm–Bonferroni step-down procedure was applied to the *p*-values obtained from the group × time interaction tests. This procedure was used to control the family-wise Type I error rate at a two-sided α level of 0.05. Both unadjusted and Holm-adjusted *p*-values were considered when interpreting the primary outcomes.

Observed means and standard deviations (SDs) were summarized for each group at baseline and 2 months. The LMMs were used to estimate within-group changes and between-group differences in change with corresponding 95% confidence intervals (CIs). The between-group difference in change was considered the principal effect estimate. To facilitate interpretation of the magnitude of the intervention effect, standardized between-group differences in change were additionally expressed as Hedges’ *g* with 95% CIs. Hedges’ *g* was calculated using the pooled baseline SD of the two randomized groups as the standardizer, with a small-sample bias correction. Percentile bootstrap 95% CIs were estimated using 20,000 participant-level resamples, stratified by randomized group. Change was defined as the 2-month score minus the baseline score. Accordingly, a positive change represented improvement in HF-related dietary behaviors, whereas a negative change represented improvement in HF symptom burden and medication adherence because higher HFSSS scores indicate greater symptom burden and lower ARMS-7 scores indicate better medication adherence.

Exploratory analyses were conducted for the four HFDBS subscales. Between-group differences in individual change scores were compared using Welch’s *t*-tests, with Holm adjustment for the four exploratory comparisons. Internal consistency was evaluated separately for each subscale at baseline and at the 2-month assessment. These analyses were considered exploratory and were not included in the family of three prespecified primary outcome tests. The results are presented in Supplementary Table S2.

Because an age imbalance was observed between groups at baseline, a post hoc sensitivity analysis was additionally performed for each primary outcome using age-adjusted LMMs estimated by restricted maximum likelihood. These models included group, time, group × time, baseline age, and age × time as fixed effects, with a participant-specific random intercept. Baseline age was centered at the overall sample mean. These analyses were considered sensitivity analyses; the primary inference regarding intervention effectiveness was based on the group × time interaction and between-group difference in change obtained from the unadjusted primary LMMs. All 60 randomized participants completed both the baseline and 2-month assessments and were analyzed in their originally assigned groups. Consequently, there were no missing primary outcome data and no imputation was required. All statistical tests were two-sided, with statistical significance set at *p* < 0.05. Values below 0.001 were reported as *p* < 0.001.

## 3. Results

### 3.1. Participant Flow and Baseline Characteristics

A total of 90 patients were approached during the recruitment process. Of these, eight were ineligible because of cognitive impairment, five did not meet the Turkish-language communication criterion, and two declined participation in the online intervention. An additional 15 eligible patients participated in the pilot phase and were not enrolled in the main randomized trial. The remaining 60 participants were enrolled and randomly allocated in a 1:1 ratio to the intervention (*n* = 30) or control group (*n* = 30). All 30 participants allocated to the intervention group received the structured online education and completed both planned telephone follow-up calls. All participants completed the 2-month follow-up assessment and were included in the analysis in their originally assigned groups. No participants were lost to follow-up, and no primary outcome data were missing (Figure 1).

Baseline demographic and clinical characteristics of the randomized participants are presented in Table 1. The mean age was 55.66 ± 5.86 years in the intervention group and 62.73 ± 13.94 years in the control group. Women accounted for 43.3% of participants in both groups. NYHA functional class was available for 18 participants (30.0%), whereas it could not be established for 42 participants (70.0%). Other baseline demographic and clinical characteristics are summarized in Table 1. Given the observed baseline difference in age between the randomized groups, an additional age-adjusted sensitivity analysis was performed.

### 3.2. Effects of the Intervention on Primary Outcomes

Changes in the three prespecified primary outcomes are presented in Table 2. Significant group × time interactions were observed for HF-related dietary behaviors, HF symptom burden, and medication adherence (all *p* < 0.001).

HF-related dietary behavior scores increased from 49.23 ± 5.89 at baseline to 61.97 ± 3.49 at 2 months in the intervention group, whereas scores remained essentially unchanged in the control group (49.27 ± 5.64 to 49.23 ± 4.70). The group × time interaction was statistically significant (F(1,58) = 100.94, *p* < 0.001). The between-group difference in change was 12.77 points (95% CI, 10.22 to 15.31), with a Hedges’ *g* of 2.18 (95% CI, 1.74 to 2.87), indicating greater improvement in HF-related dietary behaviors in the intervention group.

HF symptom burden decreased from 33.43 ± 20.96 at baseline to 14.43 ± 8.34 at 2 months in the intervention group and from 31.13 ± 18.95 to 29.40 ± 14.84 in the control group. The group × time interaction was statistically significant (F(1,58) = 22.45, *p* < 0.001). The between-group difference in change was −17.27 points (95% CI, −24.56 to −9.97), with a Hedges’ *g* of −0.85 (95% CI, −1.23 to −0.54), indicating a greater reduction in HF symptom burden in the intervention group.

ARMS-7 scores decreased from 9.70 ± 2.18 at baseline to 7.33 ± 0.55 at 2 months in the intervention group, whereas scores remained nearly unchanged in the control group (9.93 ± 3.05 to 9.90 ± 2.35). The group × time interaction was statistically significant (F(1,58) = 20.25, *p* < 0.001). The between-group difference in change was −2.33 points (95% CI, −3.37 to −1.30), with a Hedges’ *g* of −0.87 (95% CI, −1.35 to −0.50), indicating greater improvement in self-reported medication adherence in the intervention group. At the 2-month assessment, 21 of 30 participants (70.0%) in the intervention group and 6 of 30 participants (20.0%) in the control group had the minimum possible ARMS-7 score of 7, demonstrating a more pronounced floor effect in the intervention group.

After adjustment for multiple comparisons using the Holm–Bonferroni procedure, the group × time interactions remained statistically significant for all three prespecified primary outcomes: HF-related dietary behaviors (adjusted *p* < 0.001), HF symptom burden (adjusted *p* < 0.001), and medication adherence (adjusted *p* < 0.001). Thus, the statistical significance of the primary findings was maintained after controlling for multiple testing.

### 3.3. Age-Adjusted Sensitivity Analysis

Because the intervention and control groups differed in age at baseline, post hoc age-adjusted sensitivity analyses using linear mixed-effect models were performed for all three primary outcomes. After adjustment for baseline age, the estimated between-group difference in change remained statistically significant for HF-related dietary behaviors (adjusted difference, 12.93 points; 95% CI, 10.29 to 15.58; *p* < 0.001), HF symptom burden (adjusted difference, −21.41 points; 95% CI, −28.22 to −14.60; *p* < 0.001), and medication adherence (adjusted difference, −1.97 points; 95% CI, −3.01 to −0.93; *p* < 0.001). Thus, adjustment for baseline age did not alter the direction or statistical significance of the primary findings. These sensitivity analyses suggest that the observed between-group differences were not explained solely by the baseline age imbalance. Detailed results of the age-adjusted linear mixed-effect sensitivity analyses are presented in Supplementary Table S1.

## 4. Discussion

This randomized controlled trial evaluated a theory-driven nursing intervention combining Pender’s Health Promotion Model (HPM)-based structured online education with planned telephone follow-up and reinforcement in adults with HF. Compared with routine clinical care, the intervention was associated with greater improvements in self-reported HF-related dietary behaviors and medication adherence and a greater reduction in self-reported HF symptom burden over the 2-month follow-up period. Significant group × time interactions were observed for all three prespecified primary outcomes, with between-group differences in change consistently favoring the intervention group. These findings remained statistically significant after Holm–Bonferroni adjustment for multiple comparisons. The direction and statistical significance of these differences were retained in post hoc age-adjusted linear mixed-effect sensitivity analyses. Overall, the findings suggest that individualized HPM-based online education combined with planned telephone reinforcement may support multiple dimensions of HF self-management over the short term.

The improvement in HF-related dietary behaviors observed in the present study is consistent with previous evidence showing that structured educational interventions can support dietary self-management in patients with HF. Arcand et al. [17] reported that dietitian-delivered counseling reduced dietary sodium intake over 3 months compared with usual care in patients with stable HF. Similarly, Welsh et al. [18] found lower dietary sodium intake at 6 months following a theory-based intervention targeting low-sodium dietary behavior, although no significant between-group difference was observed at 6 weeks. Dunbar et al. [19] also demonstrated reductions in dietary sodium intake following patient-family education and family partnership interventions compared with usual care. Together, these findings support the potential value of structured education and continued behavioral support for dietary self-management in HF. However, direct comparisons should be made cautiously because these earlier trials primarily evaluated sodium intake, whereas the present study assessed a broader range of HF-related dietary behaviors. The HFDBS demonstrated lower internal consistency at baseline (Cronbach’s α = 0.599) than at the 2-month assessment (α = 0.819), indicating differences in the consistency of item responses across measurement occasions. Although exploratory analyses suggested improvements across all four dietary behavior subscales, internal consistency varied considerably across domains, with relatively low coefficients for several subscales (Supplementary Table S2). These findings suggest that the observed improvement in total dietary behavior scores was accompanied by changes across multiple dietary domains; however, the magnitude of these changes, particularly the large standardized effect, should be interpreted cautiously in light of the measurement limitations. Furthermore, improvements in self-reported dietary behaviors should not be interpreted as direct evidence of reduced dietary sodium intake.

Beyond dietary outcomes, the application of Pender’s Health Promotion Model in web-based patient education has also been examined by Javadzade et al. [20] in individuals with cardiovascular disease. Although the study population and intervention characteristics differed from those of the present trial, this study provides relevant context for the application of theory-informed digital education in cardiovascular care.

The reduction in self-reported HF symptom burden observed in the present study is consistent with previous evidence supporting structured education combined with continued follow-up for symptom management. In a randomized controlled trial involving 64 patients with HF, İlaslan and Özer [6] evaluated a multicomponent intervention comprising web-based education, four telephone follow-up sessions, and weekly text messages. At 12 weeks, patients receiving the intervention showed greater improvement in symptom burden and health status than those receiving routine care. Although their intervention involved more frequent follow-up and additional text messaging and differed from the present study in outcome measurement, the direction of the findings is consistent with the greater reduction in symptom burden observed in our intervention group. At a broader level, Huang et al. [21], in a systematic review and meta-analysis of 25 randomized controlled trials involving 2746 participants, found that nurse-led HF self-care interventions improved symptom burden compared with control conditions. However, the certainty of evidence for this outcome was rated as low. Thus, while the present findings are consistent with the broader evidence supporting nurse-led self-care interventions for symptom management, the observed reduction should be interpreted as an improvement in patient-reported symptom burden rather than definitive evidence of improved clinical status. Changes in medication regimens, disease severity, or other aspects of clinical management during follow-up were not systematically documented and may have influenced symptom scores. Moreover, although the between-group difference was statistically significant, its clinical importance cannot be established solely from the observed score change without an applicable, validated minimal clinically important difference threshold. Additional well-designed trials with longer follow-up and objective clinical assessments are needed to establish the consistency, clinical relevance, and durability of these effects.

The reduction in symptom burden may be partly related to the intervention’s emphasis on symptom recognition, self-management, and continued reinforcement. Participants received structured education addressing HF symptoms and their management, while the subsequent telephone contacts provided opportunities to discuss current symptoms, clarify difficulties, and reinforce relevant self-management recommendations. Evidence from earlier randomized research supports the potential value of such reinforcement: Baker et al. [22] found that a Teach-to-Goal intervention incorporating repeated telephone counseling produced greater improvements in self-care behaviors and HF-related outcomes than a brief educational intervention alone. Within the HPM framework used in the present study, attention to perceived barriers, self-efficacy, motivation, and readiness for change may also have supported engagement with symptom-management recommendations. However, these constructs and symptom-recognition skills were not measured as potential mediators. Therefore, the mechanisms underlying the reduction in symptom burden cannot be established from the present findings.

The improvement in self-reported medication adherence observed in the present study is consistent with previous evidence indicating that structured education combined with continued follow-up can support medication-taking behaviors in patients with HF. Oscalices et al. [23] found that a behavioral intervention comprising individualized discharge guidance and telephone follow-up at 7 and 30 days resulted in greater medication and non-pharmacological treatment adherence at 90 days compared with usual care. Similarly, Yu et al. [24] reported greater improvement in medication adherence among patients with HF who received a health education booklet combined with telephone follow-up than among those receiving usual care. Supporting evidence has also been reported using a different approach to adherence assessment. Granger et al. [25], in the Chronic Heart Failure Intervention to Improve Medication Adherence (CHIME) randomized trial, found that a nurse-delivered self-management intervention focusing on medication goals, medication–symptom associations, and symptom-response planning increased the likelihood of medication adherence across follow-up assessments compared with an attention-control condition, with adherence assessed using nurse-conducted pill counts. Although these interventions differed in content, intensity, follow-up duration, and adherence measurement, their findings are broadly consistent with the improvement observed in the present study and support the potential value of extending medication education beyond the initial encounter through continued reinforcement and opportunities to address medication-related difficulties. In the present study, lower ARMS-7 scores indicated better medication adherence, and the intervention group demonstrated a greater reduction in scores than the control group. However, at the 2-month assessment, 70% of intervention participants (21/30) and 20% of control participants (6/30) achieved the minimum possible ARMS-7 score of 7, indicating a floor effect, particularly in the intervention group. This concentration of scores at the lower boundary may have limited the instrument’s ability to distinguish further improvements among participants reporting high adherence. Accordingly, although the findings favor the intervention, the magnitude of the observed effect should be interpreted cautiously, and improvements in self-reported adherence should not be assumed to reflect equivalent changes in objectively measured medication-taking behavior.

Taken together, the present findings extend the existing evidence on theory-based, nurse-led approaches to HF self-management. Recent evidence indicates that technology-supported nurse-led interventions may improve self-care in patients with HF, although intervention components and delivery strategies vary considerably across studies and substantial between-study heterogeneity remains [5]. The present trial contributes to this evidence by evaluating a clearly defined nursing intervention that integrated HPM-based individualized synchronous online education with planned telephone reinforcement and by examining three complementary, prespecified primary outcomes within the same randomized design: HF-related dietary behavior, medication adherence, and symptom burden. The concurrent improvement across these behavioral and symptom-related outcomes suggests that the potential value of the combined intervention may extend across multiple dimensions of HF self-management rather than being confined to a single behavior or outcome. Although the intervention was informed by key HPM constructs, including perceived benefits and barriers, self-efficacy, and interpersonal influences, these constructs were not directly measured. Therefore, the findings cannot establish whether changes in the proposed theoretical mechanisms accounted for the observed improvements. Furthermore, because online education and telephone reinforcement were delivered as a combined intervention, their individual contributions cannot be distinguished. However, the study was not designed to determine whether improvements in dietary behavior or medication adherence contributed to the reduction in symptom burden, and no mediation analysis was performed. The findings should therefore be interpreted as evidence of the overall effect of the combined intervention rather than evidence of causal relationships among the three outcomes. By reporting intervention effects across these complementary outcomes together with confidence intervals and standardized effect estimates, this trial provides additional quantitative evidence that may inform future studies and evidence syntheses of theory-based, technology-supported nursing interventions for HF.

From a nursing practice perspective, these findings suggest that individualized synchronous online education combined with planned telephone reinforcement may provide a promising approach to supplementing face-to-face care and extending HF education beyond initial clinical encounters. Such an approach may provide opportunities to reinforce dietary recommendations, medication-taking behaviors, and symptom-management strategies after the initial educational contact. However, its feasibility and applicability may depend on patients’ access to digital technologies, digital literacy, and the availability of nursing resources to support individualized education and follow-up. Furthermore, the findings from this single-center trial with a relatively small sample and short follow-up do not establish the effectiveness of this specific intervention protocol for routine implementation. Further multicenter studies with larger and more diverse samples and longer follow-up are needed to determine its sustainability, scalability, and applicability across different HF populations and healthcare settings.

### Strengths and Limitations

This study has several strengths, including its randomized controlled design, allocation concealment until completion of baseline assessments, complete follow-up of all randomized participants, and absence of missing primary outcome data. The intervention was explicitly grounded in Pender’s Health Promotion Model and combined individualized synchronous online education with planned telephone reinforcement. Three prespecified primary outcomes representing complementary behavioral and symptom-related dimensions of HF self-management were evaluated. Reporting between-group differences in change with 95% confidence intervals and standardized effect estimates, together with Holm–Bonferroni adjustment for multiple primary outcome comparisons and post hoc age-adjusted sensitivity analyses, supports transparent interpretation of the findings. The direction and statistical significance of the intervention effects were consistent across all three primary outcomes after these additional analyses.

Several limitations should be acknowledged. First, the single-center design and relatively small sample (*N* = 60) may limit generalizability and the precision of effect estimates, particularly the large standardized effects. Eligibility requirements related to digital access and Turkish-language literacy further restrict applicability to some HF populations. Incomplete baseline information on HF severity, particularly NYHA functional class, limited assessment of clinical comparability between groups. Second, the 2-month follow-up does not establish the sustainability of the observed effects. Third, all primary outcomes were self-reported and therefore susceptible to recall and social desirability bias. The ARMS-7 floor effect at follow-up and the relatively low baseline internal consistency of the HFDBS (Cronbach’s α = 0.599), together with variable and often low subscale reliability, warrant caution when interpreting effect magnitudes. Moreover, the clinical importance of the reduction in symptom burden remains uncertain without an applicable validated minimal clinically important difference threshold. Fourth, despite randomization, baseline age differed between groups. Although age-adjusted sensitivity analyses yielded consistent findings, residual confounding cannot be excluded. Changes in medication regimens, HF severity, or clinical management during follow-up were not systematically documented. Fifth, the transition from in-person baseline assessments to electronic follow-up may have introduced a mode-of-administration effect. Participants and the intervention researcher could not be blinded after allocation, and the researcher was aware of group assignment at follow-up. Finally, the combined intervention design prevents separation of the effects of online education and telephone reinforcement, while the absence of direct measurements of HPM constructs precludes evaluation of the proposed behavioral mechanisms. Harms and unintended effects were not systematically assessed; therefore, the study cannot establish the safety of the intervention or exclude potential intervention-related adverse effects.

Despite these limitations, the randomized design, complete follow-up, and consistency of findings across the three prespecified primary outcomes support the short-term potential of the combined intervention. Future adequately powered multicenter trials should include more diverse HF populations, longer follow-up periods (6–12 months), objective or independently verified measures of dietary intake and medication adherence, and standardized assessments of HF severity and treatment changes. Component-evaluation designs and direct measurement of HPM constructs could clarify the contributions of individual intervention components and potential behavioral mechanisms. Further implementation research should examine feasibility, acceptability, resource requirements, and scalability across nursing care settings.

## 5. Conclusions

This randomized controlled trial demonstrated that Pender’s Health Promotion Model-based structured online education combined with planned telephone reinforcement was associated with greater improvement in self-reported HF-related dietary behaviors and medication adherence and a greater reduction in self-reported HF symptom burden than routine clinical care over a 2-month follow-up period. The between-group differences remained statistically significant after Holm–Bonferroni adjustment for the three prespecified primary outcomes. The direction and statistical significance of these effects were retained in post hoc age-adjusted sensitivity analyses. These findings suggest that integrating theory-based, individualized online education with continued telephone reinforcement may support multiple dimensions of HF self-management in the short term. However, the single-center design, relatively small sample, short follow-up period, and reliance on self-reported outcomes should be considered when interpreting the findings. The results reflect the combined intervention and do not establish the independent contributions of online education and telephone reinforcement or the long-term clinical significance of the observed changes. Larger multicenter trials with longer follow-up and complementary objective outcome measures are needed to determine the sustainability and broader applicability of these effects.

## Figures and Tables

**Table 1:** Baseline demographic and clinical characteristics of the randomized participants (*N* = 60).

Characteristic	Intervention (*n* = 30)	Control (*n* = 30)
**Age, years, mean ± SD**	55.66 ± 5.86	62.73 ± 13.94
**Sex,** ***n*** **(%)**		
Female	13 (43.3)	13 (43.3)
Male	17 (56.7)	17 (56.7)
**Education level,** ***n*** **(%)**		
Primary education	6 (20.0)	8 (26.7)
High school	12 (40.0)	15 (50.0)
University	12 (40.0)	7 (23.3)
**Duration of HF,** ***n*** **(%)**		
6 months–1 year	4 (13.3)	7 (23.3)
>1–<5 years	11 (36.7)	12 (40.0)
≥5 years	15 (50.0)	11 (36.7)
**NYHA class,** ***n*** **(%)**		
I	1 (3.3)	0 (0.0)
II	6 (20.0)	6 (20.0)
III	2 (6.7)	1 (3.3)
IV	1 (3.3)	1 (3.3)
Unknown	20 (66.7)	22 (73.3)
**Comorbidity other than HF,** ***n*** **(%)**		
Yes	26 (86.7)	29 (96.7)
No	4 (13.3)	1 (3.3)
**Number of medications used per day, mean ± SD**	7.06 ± 2.87	6.20 ± 2.36

Abbreviations: HF, heart failure; NYHA, New York Heart Association; SD, standard deviation; Unknown NYHA class indicates that participants were unable to report their functional class and that the classification could not be established from the available medical records, although the diagnosis of heart failure was documented.

**Table 2:** Effects of the intervention on HF-related dietary behaviors, HF symptom burden, and medication adherence based on linear mixed-effect models.

Outcome	Group	Baseline, Mean ± SD	2 Months, Mean ± SD	Within-Group Change † (95% CI)	Between-Group Difference in Change (95% CI)	Group × Time F(1, 58)	*p*	Hedges’ g (95% CI)	Holm-Adjusted *p*
**HFDBS**	Intervention	49.23 ± 5.89	61.97 ± 3.49	+12.73 (10.88 to 14.59)	+12.77 (10.22 to 15.31)	100.94	<0.001	2.18 (1.74 to 2.87)	<0.001
	Control	49.27 ± 5.64	49.23 ± 4.70	−0.03 (−1.85 to 1.79)	—	—	—	—	—
**HFSSS**	Intervention	33.43 ± 20.96	14.43 ± 8.34	−19.00 (−24.67 to −13.33)	−17.27 (−24.56 to −9.97)	22.45	<0.001	−0.85 (−1.23 to −0.54)	<0.001
	Control	31.13 ± 18.95	29.40 ± 14.84	−1.73 (−6.57 to 3.11)	—	—	—	—	—
**ARMS-7**	Intervention	9.70 ± 2.18	7.33 ± 0.55	−2.37 (−3.08 to −1.66)	−2.33 (−3.37 to −1.30)	20.25	<0.001	−0.87 (−1.35 to −0.50)	<0.001
	Control	9.93 ± 3.05	9.90 ± 2.35	−0.03 (−0.82 to 0.75)	—	—	—	—	—

† Change was calculated as the 2-month score minus the baseline score. A positive change indicates improvement in HFDBS scores, whereas a negative change indicates improvement in HFSSS and ARMS-7 scores. Hedges’ g was calculated using the pooled baseline standard deviation of the two randomized groups, with a small-sample bias correction. Corresponding 95% confidence intervals were estimated using 20,000 stratified participant-level bootstrap resamples. Holm-adjusted *p*-values account for multiple testing across the three prespecified primary outcomes. Abbreviations: CI, confidence interval; HFDBS, Heart Failure Dietary Behavior Scale; HFSSS, Heart Failure Symptom Status Scale; ARMS-7, 7-item Adherence to Refills and Medications Scale; SD, standard deviation.

## Data Availability

The de-identified participant data supporting the findings of this study are available from the corresponding author upon reasonable request, subject to applicable ethical and institutional requirements. The data are not publicly available due to privacy and ethical considerations related to participant data. No separate statistical code was generated for this study. Additional trial materials, including the educational materials used in the intervention, are available from the corresponding author upon reasonable request.

## Supplementary Materials

The following supporting information can be downloaded at: https://www.mdpi.com/article/10.3390/healthcare14213796/s1, Table S1: Age-adjusted linear mixed-effects sensitivity analyses of intervention effects on heart failure outcomes; Table S2: Exploratory HFDBS subscale analyses at baseline and two-month follow-up.

## Abbreviations

The following abbreviations are used in this manuscript:

ARMS-7	7-item Adherence to Refills and Medications Scale
CI	Confidence interval
HF	Heart failure
HFDBS	Heart Failure Dietary Behavior Scale
HFSSS	Heart Failure Symptom Status Scale
HPM	Health Promotion Model
LMM	Linear mixed-effect model
SD	Standard deviation
