# Supplementary material for: Effects of Pender’s Health Promotion Model-Based Online Education and Telephone Follow-Up on Symptom Burden, Dietary Behaviors, and Medication Adherence in Adults with Heart Failure: A Randomized Controlled Trial
Source: Healthcare (Basel). 2026 Nov 6;14(21):3796. doi: 10.3390/healthcare14213796 (PMC13763089; doi:10.3390/healthcare14213796)
Supplement: Supplementary file 1 [file healthcare-14-03796-s001.zip › healthcare-4640871-supplementary.pdf]

Supplementary Table S1. Age-adjusted linear mixed-effects sensitivity analyses of intervention effects on heart failure outcomes.

Outcome	Adjusted difference in change (95% CI)	p-value
HF-related dietary behaviors	12.93 (10.29 to 15.58)	<0.001
HF symptom burden	−21.41 (−28.22 to −14.60)	<0.001
Medication adherence	−1.97 (−3.01 to −0.93)	<0.001

Note: Estimates were obtained from linear mixed-effects models adjusted for baseline age. Adjusted differences represent between-group differences in changes from baseline to the 2-month follow-up. Higher scores indicate better HF-related dietary behaviors, whereas lower scores indicate lower HF symptom burden and better medication adherence. CI, confidence interval; HF, heart failure.

Supplementary Table S2. Exploratory HFDBS Subscale Analyses at Baseline and Two-Month Follow-Up

Panel A. Subscale scores and between-group differences in change

HFDBS subscale	Intervention T0	Intervention T1	Control T0	Control T1	Difference in change (95% CI)	Holm-adjusted p
Healthy habits	12.53 ± 2.90	15.73 ± 2.15	12.17 ± 1.98	12.83 ± 2.45	2.53 (1.51 to 3.56)	<0.001
Salt restriction	18.70 ± 3.71	23.33 ± 0.84	18.60 ± 3.85	18.13 ± 2.53	5.10 (3.57 to 6.63)	<0.001
Sugar restriction	9.43 ± 1.72	11.80 ± 0.41	9.23 ± 2.03	8.47 ± 1.72	3.13 (2.28 to 3.98)	<0.001
Prevention of fluid retention	8.57 ± 1.83	11.10 ± 1.54	9.27 ± 1.72	9.80 ± 2.02	2.00 (1.28 to 2.72)	<0.001

Panel B. Internal consistency (Cronbach's α; pooled intervention and control groups, N = 60)

HFDBS subscale	Items (n)	Baseline α	Two-month α
Healthy habits	5	0.358	0.527
Salt restriction	6	0.810	0.775
Sugar restriction	3	0.327	0.683
Prevention of fluid retention	5	0.286	0.311

Notes. Values in Panel A are mean ± SD. Change was defined as the two-month score minus baseline; positive differences favor the intervention. Between-group differences in change and 95% confidence intervals were estimated using Welch comparisons of individual change scores. Holm-adjusted p values account for four exploratory subscale comparisons; these are not the prespecified primary-outcome tests. Panel B summarizes internal-consistency estimates calculated from item-level data pooled across groups at each occasion. Several subscales showed low internal consistency; exploratory findings should not be treated as confirmatory evidence. T0, baseline; T1, two months; HFDBS, Heart Failure Dietary Behavior Scale; CI, confidence interval; SD, standard deviation.
